# Associations between maternal urinary iodine assessment, dietary iodine intakes and neurodevelopmental outcomes in the child: a systematic review

**DOI:** 10.1186/s13044-021-00105-1

**Published:** 2021-06-07

**Authors:** Anna M. Monaghan, Maria S. Mulhern, Emeir M. McSorley, J. J. Strain, Matthew Dyer, Edwin van Wijngaarden, Alison J. Yeates

**Affiliations:** 1 Nutrition Innovation Centre for Food and Health, Ulster University, Coleraine, Northern Ireland; 2 The Department of Community and Preventive Medicine, University of Rochester School of Medicine and Dentistry, Rochester, NY USA

**Keywords:** Urinary iodine assessment, Dietary iodine intakes, Pregnancy, Neurodevelopment, Offspring

## Abstract

**Objective:**

Mild to moderate iodine deficiency during pregnancy has been associated with adverse neurodevelopmental outcomes in offspring. Few research studies to date combine assessment of urinary iodine (UIC and/or ICr), biomarkers that best reflect dietary intake, with reported dietary intake of iodine rich foods in their assessment of iodine deficiency. Thus, a systematic review was conducted to incorporate both these important measures.

**Design:**

Using PRISMA guidelines, a comprehensive search was conducted in three electronic databases (EMBASE®, MedLine® and Web of Science®) from January 1970–March 2021. Quality assessment was undertaken using the Newcastle Ottawa Scale. Eligible studies included reported assessment of iodine status through urinary iodine (UIC and/or ICr) and/or dietary intake measures in pregnancy alongside neurodevelopmental outcomes measured in the children. Data extracted included study author, design, sample size, country, gestational age, child age at testing, cognitive tests, urinary iodine assessment (UIC in μg/L and/or ICr in μg/g), dietary iodine intake assessment and results of associations for the assessed cognitive outcomes.

**Results:**

Twelve studies were included with nine reporting women as mild-moderately iodine deficient based on World Health Organization (WHO) cut-offs for urinary iodine measurements < 150 μg/l, as the median UIC value in pregnant women. Only four of the nine studies reported a negative association with child cognitive outcomes based on deficient urinary iodine measurements. Five studies reported urinary iodine measurements and dietary intakes with four of these studies reporting a negative association of lower urinary iodine measurements and dietary iodine intakes with adverse offspring neurodevelopment. Milk was identified as the main dietary source of iodine in these studies.

**Conclusion:**

The majority of studies classified pregnant women to be mild-moderately iodine deficient based on urinary iodine assessment (UIC and/or ICr) and/or dietary intakes, with subsequent offspring neurodevelopment implications identified. Although a considerable number of studies did not report an adverse association with neurodevelopmental outcomes, these findings are still supportive of ensuring adequate dietary iodine intakes and urinary iodine monitoring throughout pregnancy due to the important role iodine plays within foetal neurodevelopment. This review suggests that dietary intake data may indicate a stronger association with cognitive outcomes than urinary iodine measurements alone. The strength of this review distinguishes results based on cognitive outcome per urinary iodine assessment strategy (UIC and/or ICr) with dietary data. Future work is needed respecting the usefulness of urinary iodine assessment (UIC and/or ICr) as an indicator of deficiency whilst also taking account of dietary intakes.

**Supplementary Information:**

The online version contains supplementary material available at 10.1186/s13044-021-00105-1.

## Introduction

Iodine is an essential mineral required for the production of the thyroid hormones triiodothyronine (T3) and thyroxine (T4) [1]. Most countries set iodine requirements for the general adult population at 150 μg/d, based on WHO recommendations, with this increasing to 250 μg/d during pregnancy [2, 3]. Requirements increase mainly via the need for increased thyroid hormone production and because the foetus is entirely dependent on the mother [4, 5]. Based on these increased requirements and the important role iodine plays in foetal neurodevelopment, pregnant women are at an increased risk of deficiency, particularly through poor consumption of iodine rich foods in the Western diet [6]. Iodine is abundant in fish products particularly white fish varieties, such as haddock, which can contain up to 400mcg per 100 g serving [7]. Different fish species contain varying iodine concentrations [7]. Milk and dairy products also contribute significantly to iodine concentrations, with these sources, owing to their frequent consumption, being one of the main contributors to dietary iodine intake [7]. The actual amount of iodine in food varies significantly owing to farming practices including the use of iodophor salts for cleaning cow udder, soil content and season and as such it can be difficult to estimate dietary intake of iodine from various food sources [8]. Globally, pregnant women have been identified as a vulnerable group regarding iodine deficiency with studies often reporting average dietary intakes throughout pregnancy well below the recommended intake of 250 μg/d [2, 9]. A contributing factor to this deficiency may be a lack of both awareness and knowledge regarding the most significant dietary contributors to iodine, and thereby reflected in the urinary assessment [10]; indeed, poor knowledge on iodine has been observed in pregnant women in many countries including the UK, Australia and Norway [11–13].

Clinical definition of iodine deficiency during pregnancy is determined by measurement of urinary iodine, with United Nations Children’s Fund (UNICEF) and WHO guidelines stating that concentrations < 150 μg/L are indicative of deficient iodine intake with this threshold referring to the median UIC value in a population of pregnant women [1, 9]. Given iodine’s crucial role in growth and cognitive development, it has been concluded that severe iodine deficiency during pregnancy results in numerous adverse outcomes in offspring including abnormal cognitive functioning, clinically manifested as cretinism in children [4].

Urinary iodine assessment provides an indication of recent iodine intakes owing to over 90% of ingested iodine being excreted in the urine [4, 10]. The value of dietary assessment is that it can provide insight into longer term intake of iodine rich foods e.g., using Food Frequency Questionnaires (FFQ’s) or Dietary Recall [11]. When used together, urinary iodine assessments (UIC and/or ICr) along with dietary data may provide a more robust identification of iodine sufficiency or deficiency and thereby represent an ideal approach to investigating potential associations with neurodevelopmental outcomes in offspring. However, research studies investigating iodine deficiency tend to investigate either UIC/ICr or dietary intakes, failing to combine these two important measures with often one or the other chosen. Indeed, a recent review by Nazarpour et al.*,* in 2019 only focused on UIC measurements omitting the role of dietary iodine intakes [14].

Therefore, this review aimed to evaluate the evidence for associations between iodine intakes and neurodevelopmental outcomes in the offspring of pregnant women, focusing on iodine intakes measured by both maternal urinary iodine assessment (UIC and/or ICr) and dietary iodine intakes.

## Methods

The Preferred Reporting Items for Systematic Reviews and Meta-Analyses (PRISMA) guidelines for systematic reviews were followed [15]. The review was registered in PROSPERO with identified ID CRD42019139554. Quality assessment was undertaken using the Newcastle Ottawa Scale (NOS) [16].

### Search strategy and eligibility criteria

A comprehensive search was conducted in three electronic databases (EMBASE®, MedLine® and Web of Science®). All searches were carried out using terms related to iodine nutrition combined with search terms for neurodevelopment respectively **(**Additional File 1**).** In addition to the databases searched, bibliographies of key studies were also hand searched for other relevant publications, although this yielded no additional results.

Articles were included if published between January 1970–March 2021 and of any study design, if the full paper (in English) was able to be sourced and if there were available cognitive, dietary and/or urinary iodine (UIC and/or ICr) data. Articles were selected from 1970 onwards owing to the work conducted by Pharoah et al.*,* who initially reported the important link between iodine deficiency during pregnancy and neurological damage in the foetus [17]. Initial screening for excluded articles was conducted independently by reviewer AM, with excluded articles based on those 1) focused solely on iodine supplementation 2) no urinary iodine assessment (UIC and/or ICr) or dietary data recorded 3) no cognitive testing conducted and 4) animal studies. The authors had initially chosen to focus on the natural dietary forms of iodine, choosing to exclude iodine supplementation studies. Studies that mentioned “accidental supplementation” of iodine in the diet i.e., where supplementation was not enforced (as part of a trial) were considered, with the focus on the habitual, unmodified diet of pregnant populations in our review. Screening of abstracts, and searching of reference lists was conducted by AM, with final selected full texts independently reviewed by 3 reviewers (AM, MD &AY).

### Study selection and data extraction

All database search results were exported to RefWorks and duplicate records removed. Articles were then initially screened, with any duplicates and/or articles not matching the inclusion criteria omitted (AM). **(**Table 1**).** After applying the inclusion/exclusion criteria, full texts for each article were obtained and subsequently assessed for eligibility. Reference lists of relevant studies were also hand searched for further eligible papers with these then screened independently by three reviewers (AM, MD and AY). Upon implementation of the inclusion and exclusion criteria the relevant studies for inclusion in this systematic review were of a prospective cohort/longitudinal design, with this reflected in the quality assessment conducted using the relevant Newcastle Ottawa Scale (NOS) assessment strategy [16].

**Table 1:** PICOS criteria for inclusion and exclusion of studies

	Inclusion criteria	Exclusion criteria
Population	Pregnant women and their children.	Studies including women of childbearing age but not during pregnancy and/or post-natally will be excluded.
Intervention	Assessment of dietary contributors to iodine status during pregnancy and neurodevelopment outcomes in offspring including natural dietary sources and “accidental” supplementation e.g., women taking supplement prior to/not as part of the study.	Any articles involving intentional dietary supplementation of iodine will be excluded.
Comparison	Pregnant women with low iodine status during pregnancy and/or associations with neurodevelopment in offspring.	Studies that have no detail on iodine status and/or cognitive outcomes during pregnancy on offspring.
Outcomes	The main dietary contributors to iodine status during pregnancy and/or associations with neurodevelopment in offspring.	Studies that did not measure child cognitive/neurodevelopment outcomes and/or detail urinary iodine measurements or dietary data.
Study Design	All study types e.g., Prospective Cohort, Observational studies, and cross-sectional studies.	Animal study, in vitro study, drug study, chemical interaction study, laboratory study, food technology study, cell culture study, method development paper, Fortification studies, Supplementation trials, research policy/ policy making, proof of concept, letters, editorials, systematic reviews and meta-analyses, commentaries, studies not published in the English language.

The following data were extracted for each included study: study author, study design, sample size (*n*=), country, gestational age at urine sample collection and/or dietary assessment, child age at testing, cognitive tests, urinary iodine assessment strategy (UIC in μg/L and/or ICr in μg/g), dietary assessment and measures of child neurodevelopment.

### Quality assessment

The quality assessment of the 12 included eligible studies was conducted through the Newcastle-Ottawa scale (NOS), which assessed the areas of selection, comparability, and outcome. Using a predefined star scoring system, each of the studies were independently assessed, with a higher score subsequently indicative of higher quality research, up to a maximum of 9 stars [16].

## Results

**Fig. 1:**
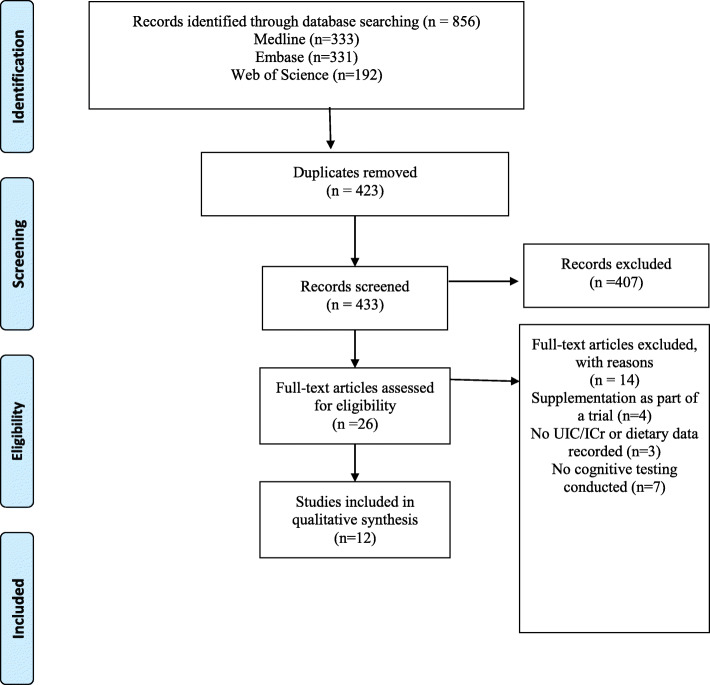
Preferred Reporting Items for Systematic Reviews and Meta-Analyses flow diagram (PRISMA) for associations between maternal urinary iodine assessment, dietary iodine intakes and neurodevelopmental outcomes in the child

The PRISMA flow diagram outlining the study selection process and number of studies at each stage of review are shown in Fig. 1. The initial electronic database search yielded a total of 856 articles (331 EMBASE, 333 Medline and 192 Web of Science) after which 423 duplicates were removed, leaving a total of 433 remaining for review. Applying the exclusion/inclusion criteria at this point, the first screening of titles and abstracts, resulted in a total of 407 article texts being removed. Following a second screening of these full-text articles, a final number of 12 relevant publications were included. This process resulted in studies of only a prospective cohort/longitudinal design being included within this systematic review. Following quality assessment for cohort studies using the Newcastle Ottawa Scale (NOS), all of the included studies, with the exception of Hynes et al.*,* (2017) scored a maximum of 9 stars [18]. Hynes et al.*,* (2017) scored 8 stars, with their “selection” score marked lower than the other included studies owing to their findings being based on previously assessed cohorts [18, 19]. Thus, overall, the quality of the included studies within this systematic review were classified as high.

Table 2 details the included studies (*n* = 12) which investigated associations between maternal urinary iodine measurements (UIC and/or ICr) and child cognitive outcomes whilst Table 3 details the included studies (*n* = 5) which focused on associations between dietary intakes and cognitive outcomes. Five of the twelve studies incorporated both urinary iodine (UIC and/or ICr) and dietary intake measures, and, therefore, are reported in both Tables 2 and 3:

**Table 2:** Characteristics and summary of findings of *n* = 12 studies included investigating associations between maternal urinary iodine assessment and child cognitive outcomes

Author	Country	Design	N	Gestational age at urine collection	Child age at testing	Cognitive tests	Urinary iodine assessment UIC and/or ICr	Association to cognitive outcomes	Quality
Bath, S.C. et al., 2013 [6]	UK	Prospective cohort	1040 mother child pairs	~ 13 weeks gestation	8–9 years	WISC; Neale Analysis of Reading Ability.	ICPMS: 91.1 μg/L UIC & ICr: 110 μg/g	Offspring of women with ICr < 150 μg/g had scores in lowest quartile (OR 1·58, 95% CI 1·09–2·30; *p* = 0·02), reading accuracy (1·69, 1·15–2·49; *p* = 0·007), and reading comprehension (1·54, 1·06–2·23; p = 0·02)	9
Markhus, M.W. et al., 2018 [20]	Norway	Prospective cohort	851 mother child pairs	23.7 weeks	6.1, 12.2 and 18.4 months	BSID-III	ICPMS: 78 μg/L *n* = 155 women reported supplement use with UIC higher 92 μg/L	UIC < 100 μg/L associated with poorer receptive (*p* = 0.025) and expressive language skills (*p* = 0.002). Iodine-containing supplements was associated with lower gross motor skills (b = −0.18, 95% CI = − 0.33, − 0.03, *p* = 0.02),	9
Van Mil, N.H. et al., 2012 [21]	The Netherlands	Prospective cohort	1156 mothers and 692 children	13.2 weeks	4	BRIEF-P	Ammonium persulfate: 203 μg/g ICr (median) Supplement use in 27.1% of women with urinary iodine <10th percentile and 30.8% >10th percentile	Offspring of women with low urinary iodine scores had higher scores on the problem scales of inhibition [b = 0.05 (95% CI: 0.01, 0.10), P = 0.03] and working memory [b = 0.07 (95% CI: 0.02, 0.12), *P* = 0.003].	9
Hynes, K.L. et al., 2013 [19]	Australia	Longitudinal follow up	433 mothers and 228 offspring	Mean 24.6 weeks (8–41 weeks range)	9	NAPLAN and SARIS tests	Kolthoff method: Median UIC 81 μg/L 71.1% mothers had UIC < 150 μg/L 29.9% mothers had UIC > 150 μg/L	Maternal UIC < 150 μg/L had offspring with 10% reductions in spelling (95% CL 68.0 to 14.3; *p =* 0.003), 7.6% in grammar (95% CI 60.2 to 1.7, *P* = 0.038), and 5.7% in English-literacy (95% CI, 0.63 to 0.03; *P* = 0.034) performance compared to maternal UIC > 150 μg/L.	9
Hynes, K.L. et al., 2017 [18]	Australia	Longitudinal follow up	449 mothers and 266 offspring	Mean 23.7 weeks (6–41 weeks)	13–14 years	NAPLAN tests	Kolthoff method: Median UIC 83.2 μg/L	Maternal UIC < 150 μg/L had offspring exhibiting persistent reductions in spelling from Year 3 (95% CL −65.1 to −17.6; *p* = 0.001)) to Year 9 (95% CL − 57.0 to − 6.2, *p* = 0.015)) compared to maternal UIC ≥ 150 μg/L.	8
Abel, M.H. et al., 2017 [22]	Norway	Prospective cohort	53′360 mother-child pairs (19′086 for ADHD score)	18 (*n* = 1950 for UIC measurements)	9.9 (median)	ADHD Rating Scale	ICPMS: Median UIC 61 μg/L (n = 1950 for non-supplement users); 86 μg/L (*n* = 988 for supplement users)	NS	9
Abel, M.H., et al., 2018 [23]	Norway	Prospective cohort	39,471 mother child pairs	0–22 (wk. 18 for UIC, *n* = 2001)	8	CCCS; Vineland Adaptive Behaviour Scale; Performance tests for reading, writing and maths.	ICPMS: Median UIC 67 μg/L; 95 μg/L in supplement users and 59 μg/L in non-supplement users.	NS	9
Murcia, M. et al., 2017 [24]	Spain	Prospective cohort	2644 women recruited and 1803 children	13.5	4.8	MSCA	Paired-ion reversed-phase HPLC: 123 μg/L UIC (median) and ICr 151 μg/g	NS	9
Zhou, S.J. et al., 2018 [25]	Australia	Prospective cohort	699 mother child pairs	< 20 and 28	19.5 months	BSID-III	Sandell-Kolthoff UIC 186 μg/L	NS	9
Cromie, K.J. et al., 2020 [26]	UK	Prospective cohort	6955 mothers	26–28 weeks	8–12 years	ASD Diagnosis through CTV3 read code	ICPMS: Median (inter-quartile range) UIC was 76 μg/L (46, 120) and ICr was 83 μg/g (59, 121)	NS	9
Ghassabian, A. et al., 2014 [27]	The Netherlands	Prospective cohort	1525 mother-child pairs	< 18 weeks gestation	6 years	Non-verbal IQ and language comprehension	Ceri-arsenite reaction: median UIC creatinine = 229.6 μg/g (whole sample) < 150 μg/g median was 119.3 μg/g (12.3%) and > 150 μg/g was 322.9 μg/g	NS	9
Threapleton, D.E. et al., 2020 [28]	UK	Prospective cohort	6971 mothers5745 children	26–28 weeks gestation	4–7 years	EYFS, phonics, and KS1 learning outcomes/social/behavioural difficulties, and sensorimotor control	Jaffe reaction: Median (interquartile range) UIC was 76 μg/L (46, 120), and ICr was 83 μg/g (59, 121).	NS	9

*UK* United Kingdom, *WISC* Wechsler Intelligence Scale for Children, *UIC* Urinary Iodine Concentration, *ICr* Iodine Creatine Ratio, *ICPMS* Inductively Coupled Plasma Mass Spectrometry, *CI* Confidence Intervals, *NS* No Significance, *BSID-III* Bayley’s Scale of Infant Development, *BRIEF-P* Behavior Rating Inventory of Executive Function, *NAPLAN* National assessment program-Literacy and Numeracy, *SARIS* Student Assessment and Reporting Information System, *ADHD* Attention-Deficit Hyperactivity Disorder, *CCCS* The Children’s Communication Checklist-short, *IQ* Intelligence Quotient, *MSCA* McCarthy Scales of Children’s Abilities, *HPLC* High Performance Liquid Chromatography, *ASD* Autism Spectrum Disorder, *EYFS* Child school achievement Early Years Foundation Stage, *KS1* Key Stage One

**Table 3:** Characteristics and summary of findings of studies (*n* = 5) investigating associations between maternal dietary iodine intakes and child cognitive outcomes

Author	Country	Design	N	Gestational age at dietary assessment	Child age at testing	Cognitive tests conducted	Dietary measurement	Association to cognitive outcomes	Quality check
Abel, M.H. et al., 2017 [22]	Norway	Prospective cohort	77,164 mother child pairs at	22 weeks	9.9 (median)	ADHD Rating Scale	FFQ Median 121 μg/d *contributed to by Milk, yoghurt, eggs, fish.	Iodine intake < 200 μg/d, associated with higher child ADHD symptom scores (*p* < 0.001).	9
Abel, M.H. et al., 2018 [23]	Norway	Prospective cohort	39,471 mother child pairs	0–22	8	CCCS; Vineland Adaptive Behaviour Scale; Performance tests for reading, writing and maths.	FFQ: Median intake from food 122 μg/d Milk/Yoghurt (47%) Lean fish (14%), Egg (4%), Fatty fish (4%), Other foods (17%) and drinking water (2%)	Low dietary iodine intake associated with poorer language(*p* = 0.013), reading (*p* = 0.019), and writing skills (*p* = 0.004). Increased likelihood of special needs education (*p* = 0.042)-all in non-supplement users	9
Murcia, M. et al., 2018 [24]	Spain	Prospective cohort	2644 women recruited and 1803 children.	10–13 and 28–32	4.8	MSCA	FFQ: 161 μg/d (mean iodine intake) Milk, yoghurt, cheese. 45.8% consumed iodized salt and 34.2% a supplement containing iodine.	Dietary iodine was inversely associated with motor scores and milk, but not other dairy products or seafood consumption (beta: −1.36; 95%CI −2.12 to −0.61; per one daily milk serving).	9
Zhou, S.J. et al., 2018 [25]	Australia	Prospective cohort	699 mother child pairs	< 20 and 28	19.5 months	BSID-III	IFFQ mean total iodine intake 309 μg/d and 150 μg/d when supplements were excluded. Fortified bread, accidental supplement use and iodized salt.	Maternal iodine intake in the lowest (< 220 μg/day) or highest (≥391 μg/day) quartile was associated with lower cognitive, language, and motor scores OR’s 2.7 (95% CI: 1.3, 5.6) to 2.8 (95% CI: 1.3, 5.7))	9
Van Mil, N.H. et al., 2012 [21]	Netherlands	Prospective cohort	1156 mothers and 692 children	13.2 (median)	4	BRIEF-P	FFQ consumption of bread [b = 0.61 (95% CI: 0.27, 0.95), P < 0.001] and eggs (b = 1.87 (95% CI: 0.13, 3.62), *P* = 0.04] was associated with higher urinary iodine.	NS	9

*ADHD* Attention-Deficit Hyperactivity Disorder, *NS* No significance, *FFQ* Food Frequency Questionnaire, *CCCS* The Children’s Communication Checklist-short, *CI* Confidence Intervals, *MSCA* McCarthy Scales of Children’s Abilities, *BSID-III* Bayley’s Scale of Infant Development, *BRIEF-P* Behavior Rating Inventory of Executive Function

### Associations between urinary iodine assessment and cognitive outcomes

As shown in Table 2, all 12 studies which investigated associations between maternal urinary iodine measures and child cognitive outcomes, were of a prospective cohort/longitudinal design [6, 18–28]. The included studies spanned a range of countries including Norway (*n* = 3), UK (*n* = 3), Spain (*n* = 1), The Netherlands (*n* = 2) and Australia (*n* = 3) [6, 18–28]. Five of these studies tested children under the age of 8 years [20, 21, 24, 25, 27], whilst the remaining seven assessed children after the age of 8 years [6, 18, 19, 22, 23, 26, 28]. Further, six of the studies tested maternal urinary iodine before 22 weeks gestation [6, 21–27], five at a gestational age > 22 weeks [18–20, 22–28], and one undertook testing across both time points [25]. Seven of the twelve studies showed no association between maternal urinary iodine assessment (UIC and/or ICr) and child cognitive outcomes [22–28], with the remaining five studies reporting better cognitive outcomes in children of mothers with higher urinary iodine [6, 18–21].

Seven of the studies reported urinary iodine through UIC measurements with only Van Mil et al.*,* (2012) reporting iodine creatine ratio (ICr) [18–23, 25, 27]. The remaining four studies reported both UIC and ICr [6, 24, 26, 28]. All studies reported UIC in μg/L and/or ICr in μg/g [6, 18–28]. Nine of these studies classified women as mildly iodine deficient based on the median values reported in accordance with the WHO classification of < 150 μg/l as deficient [6, 18–20, 22–24, 26, 28]. Pregnant women in the remaining three studies by Van Mil et al.*,* (2012), Ghassabian et al.*,* (2014) and Zhou et al.*,* (2018) were classified in the sufficient range [21, 25, 27]. None of the reported studies found women to be in either the above requirements and/or excessive, ranges [6, 18–28].

Women with a UIC < 150 μg/L were more likely to have children with persistent reductions in spelling compared to those offspring of mothers with UIC > 150 μg/l as found by Hynes et al.*,* (2017), and assessed via National assessment program-Literacy and Numeracy (NAPLAN) tests [18]. Further, Hynes et al.*,* (2013), reported that offspring of women with UIC < 150 μg/l had reductions in spelling, grammar and English literature compared to a UIC > 150 μg/l, as examined through the same cognitive tests [19]. Markhus et al.*,* (2018), who also reported UIC measurements, found that offspring of women with UIC < 100 μg/l had poorer receptive and expressive language skills, assessed via Bayley’s Scale of Infant Development, third edition (BSID-III) [20]. Bath et al.*,* (2013) was the only study which reported both UIC and ICr and which found an association between ICr recorded and cognitive outcomes, with those offspring born to mothers with an ICr < 150 μg/g obtaining lower scores for IQ assessment, as examined through Wechsler Intelligence Scale for Children (WISC), and Neale’s analysis of reading ability [6]. Interestingly, Van Mil’s et al.*,* (2012) study, which classified women as sufficient based on a median ICr of 203 μg/g, reported that offspring of mothers with low urinary iodine had negative cognitive outcomes including higher scores on problem scales of inhibition and working memory, assessed via Behavior Rating Inventory of Executive Function (BRIEF-P) [21]. The remaining seven studies reported no association of iodine intakes with cognitive outcomes irrespective of whether UIC and/or ICr was reported [22–28]. As mentioned previously, studies were focused on natural sources of dietary iodine, with those that recorded accidental supplementation included. Only four of the 12 included studies (Markhus et al.*,* 2018; Van Mil et al.*,* 2012; and Abel et al.*,* 2017; 2018), reported urinary iodine measurements and accounted for both supplemental and non-supplemental users [20–23]. With the exception of the study by Van Mil *et.al.,* (2012), all of these studies still reported a deficient urinary iodine measurement based on urinary iodine assessment [21]. A higher urinary iodine value was recorded for supplement users in all these studies than those noted for non-supplement users [20–23].

### Associations between dietary iodine intake and cognitive outcomes

As shown in Table 3, five of the included studies in this systematic review investigated dietary iodine intakes and associations with cognitive outcomes [21–25]. The included studies spanned a range of countries including Norway (*n* = 2), Spain (*n* = 1), Australia (n = 1) and The Netherlands (*n* = 1) [21–25]. Reporting of maternal dietary measures was conducted through a Food Frequency Questionnaire (FFQ) for all five studies, although reporting of intake did differ, with Van Mil et al.*,* (2012), reporting main food items [21], and the other studies providing a combination of % amount, μg/d and/or specific food items [22–25]. The five studies assessed children at a range of ages, with three testing children under the age of 5 years [21, 24–26]; whilst Abel et al.*,* in both 2017 and 2018 assessed children after the age of 8 years [22, 23]. Further, three of the studies tested maternal UIC before 22 weeks gestation (Van Mil et al.*,* 2012 and Abel et al.*,* 2017;2018) [21–23], with Murcia et al.*,* (2017) and Zhou et al.*,* (2018) reporting across both the 1st and 3rd trimesters [24, 25]. The use of “accidental” supplement use was only acknowledged by both Murcia et al.*,* (2017) and Zhou et al.*,* (2018) [24, 25], whilst also reporting natural dietary sources of iodine. Of the five studies, only Van Mil et al.*,* (2012), showed no association between maternal dietary intakes and child cognitive outcomes [21], with the remaining four studies reporting an association whereby low dietary iodine intakes resulted in negative cognitive outcomes [22–25]. The majority of studies concluded that dairy produce, in particular milk, was the main contributor to dietary iodine intakes [22–24]. Other food items identified in the included articles are indicative of advancements within food processing e.g., fortification, with cereal products contributing to iodine intakes, as reported by Van Mil et al., (2012) and Zhou et al.*,* (2018) [21, 25]. It is important to note that both these studies occurred in countries where salt iodisation is mandatory (Australia and The Netherlands), which could subsequently have influenced the results reported [21, 25]. Abel et al., (2018) reported a significant relationship between low dietary iodine intake from milk and dairy produce in the mother, with poorer language, writing and reading skills in the child, as assessed through Children’s Communication Checklist (CCCS), Vineland Adaptive Behaviour Scale and performance tests for reading, writing and maths [23]. Moreover, the Abel et al., (2017) study on Attention Deficit Hyperactivity Disorder (ADHD) reported that iodine intakes from food at < 200 μg/d were associated with higher child ADHD symptom scores using the ADHD Rating Scale [22]. Findings from Murcia et al., (2018) illustrate that low maternal dietary intake of milk (< 100 g/d) was associated with offspring having poorer motor and general functioning skills, identified through testing using the McCarthy Scales of Children’s Abilities (MSCA); although this was not observed with other iodine-rich foods such as other dairy produce and fish [24]. Zhou et al.*,* (2018) indicated the need for a delicate balance required regarding consumption of iodine rich foods, with intakes at both the lower and upper end of the spectrum (< 220 μg/d or > 391 μg/d) associated with lower cognitive, language and motor skills as assessed through Bayley’s Scale of Infant Development (BSID-III) [25]. Unlike the association found between urinary iodine measurements and cognitive outcomes, Van Mil’s *et.al.,* (2012) work assessed using Behaviour Rating Inventory of Executive Function (BRIEF-P), reported no association between impaired executive functioning in offspring and dietary iodine intakes [21]. As no other dietary measurements were reported, we are unable to compare the use of FFQ to other dietary instruments, such as 24-h recall, in order to conclusively determine whether it is potentially the best dietary assessment to use.

### Associations between urinary iodine assessment (UIC and/or ICr), dietary intakes and cognitive outcomes

Five of the included studies reported both urinary iodine measurements and dietary intakes with only Abel’s et al.*,* work in both 2017 and 2018, alongside Murcia et al.*,* (2018) classifying women as deficient based on both urinary iodine assessment and dietary intakes [22–24] However, an association with cognitive outcomes was only found in these three studies based on dietary data alone and not from the urinary iodine assessment method [22–24].

## Discussion

This review indicates that the majority of pregnant women from included studies were classified as mild-moderately iodine deficient with subsequent offspring neurodevelopmental implications identified [6, 18–20, 22–24, 26, 28]. Although a considerable number of our studies did not report an association with neurodevelopmental outcomes, these findings are still supportive of ensuring adequate dietary iodine intakes and urinary iodine monitoring throughout pregnancy due to the important role iodine has in foetal neurodevelopment [22–28]. Further, in studies investigating dietary data, milk was highlighted as the main dietary contributor to iodine intake in the majority of studies [22–24].

An association between urinary iodine measurements and cognitive outcomes in offspring was observed in five of the included studies. These studies concluded that low urinary iodine measurements in pregnant women, assessed during the 1st to 2nd trimesters (range of 9–24.6 weeks), were associated with poor neurodevelopmental outcomes such as lower IQ scores and impaired executive functioning [6, 18–21]. The work conducted by the remaining studies reported no association between maternal urinary iodine measurements and adverse cognitive outcomes in the offspring [22–28]. The lack of association in these studies could be explained by the different types of cognitive testing conducted such as Abel’s et al.*,* (2018) work focusing on ADHD, thereby potentially indicative that iodine may not play as critical a role in this cognitive outcome compared to those which reported an association [23]. Therefore, these findings highlight that depending upon the type of cognitive tests implemented, due to the different domains assessed, different associations can be recorded. It is difficult to compare the studies with respect to their cognitive outcomes owing to heterogeneity amongst the included studies. Thus, identification of the most suitable cognitive test(s) to illustrate the critical role iodine has in neurodevelopment should be focused upon and made cohesive across the pregnancy and infancy testing stages.

Moreover, the different tests used to account for maternal urinary iodine, either through UIC and/or ICr should be acknowledged, with both having strengths and limitations which may have impacted the outcome observed. Urinary iodine as UIC is an easily obtainable indicator of iodine intakes amongst a population and given that the majority of iodine absorbed in the body is excreted in the urine, it is reflective of recent intake [29]. Although UIC is the most common indicator to assess population iodine intakes there are weaknesses of using UIC independently; notably that there is a high day-to-day variability of iodine intake and this measurement only reflects recent and not long-term intake [29]. Additionally, UIC does not consider thyroid function which could potentially impact urinary iodine and overall iodine status [29]. Focusing on pregnancy, where there are numerous additional demands placed on the mother, including urinary output, ICr is considered to be a better measurement of iodine intake than UIC alone because it accounts for urine dilution, as observed during pregnancy [30]. A consensus on the best method to adopt in assessing urinary iodine is yet to be conclusively decided, with both UIC and ICr having limitations and strengths, as outlined, and these should be considered when measuring iodine intake.

As reported, five of the included studies investigated both urinary iodine assessment and dietary intakes, with four of these reporting an association to neurodevelopment based on dietary data alone and not urinary measurements, despite pregnant women being classified as mild-moderately iodine deficient based on their urinary measurements [22–25]. Thus, when viewed together diet appears to have a greater association with cognitive outcomes, although urinary iodine assessment is important independently as a reflection of dietary deficiency alongside short-term/recent intake. Moreover, there are limitations associated with FFQ including the reliance on accurate and up-to-date food composition tables, misreporting from participants and the difficulty in assessing miscellaneous intake such as through iodized salt and associated fortified food items [10]. As mentioned, as the included studies did not use another type of dietary assessment, such as 24-h recall, we are unable to conclude whether this is the best measurement to use. Therefore, future work needs to respect the usefulness of urinary iodine assessment (UIC and/or ICr) as an indicator of deficiency but place greater emphasis on different dietary measurements owing to the identified link to longer-term consequences such as neurodevelopment as outlined. As mentioned, it is difficult to compare the studies with their cognitive outcomes owing to heterogeneity amongst the included studies.

Specific focus on the age of child testing illustrates an association with child neurodevelopmental outcomes, with the included studies conducted on offspring ranging from 6 months to ~ 10 years [6, 18–28]. Behavioural, motor and language tendencies develop before the age of 8 years, with five of the included studies in this review testing offspring under the age of 8 years of age [20–22, 24, 25, 27]. Language ability of children, both receptive and expressive, is developed before the age of 8 years, with the testing conducted as part of these studies*,* indicative of the consequences of poor iodine nutrition during pregnancy on this developmental outcome [31]. The remaining seven included studies undertook testing after the age of 8 years of age [6, 18, 19, 22, 23, 26, 28], with children’s reading, writing and overall motor ability deemed to be more developed at this time point [32].Thus, these findings evidently show that testing of children across a plethora of age ranges, from infancy to pre-puberty, is important in illustrating the wide spectrum of neurodevelopmental issues that can persist into childhood owing to inadequate iodine nutrition during pregnancy. However, given both the range of ages and the differing cognitive testing conducted assessing different cognitive domains, within the included studies of this review, it is important to highlight that this may be a limitation potentially contributing to why no overall association is confirmed.

As mentioned, 5 out of the 12 studies reported dietary iodine intakes with dairy produce, in particular milk [22–24], identified as the main dietary source of iodine, concurring with common dietary advice regarding milk and dairy sources, owing to their frequent consumption, as the main sources of iodine in the diet [7]. The actual amount of iodine in food varies significantly and as such it can be difficult to ascertain if sufficient iodine is available in the diet [10]. Other food items identified in the included articles are indicative of advancements within food processing such as fortification e.g., cereal products [21], as seen in Van Mil’s et al. study (2012) [21]. Although not mandatory in the UK, a Universal Salt Iodisation (USI) programme whereby salt, typically in the form of potassium iodide, is added at the manufacturing stage is actively encouraged to help mitigate inadequate consumption of iodine rich foods [33]. Interestingly, none of the included studies mentioned goitrogens, goitrogenic substances and/or the inclusion of specific goitrogenic foods in the diet e.g., cassava, which are known to impact the availability of iodine from the diet [33]; thus, future consideration should be given to this important dietary factor regarding impacting the iodine status of study participants. Renewed public health policies on dietary sources alongside work with food manufacturers should be encouraged to help promote the importance of iodine consumption in both the pre-conception and pregnancy stages. As mentioned, the authors took the decision to exclude supplementation articles but did account for studies which may have had “accidental” supplementation either through consumption of miscellaneous items fortified with iodised salt and/or if the mothers were consuming a supplement containing iodine independently of the research study. Our findings did not report any significant benefits regarding the offspring of mothers who consumed supplemental iodine in this context, although as expected a slightly higher urinary iodine measurement was recorded [20–23].

Limitations of diet recall such as memory lapses and bias need to be appreciated when interpreting dietary data but overall, it does provide insight into longer-term consumption and significantly as reported in our studies, a relationship to cognitive development [10, 13]. Work conducted in various countries including the UK, Norway and Australia concluded that knowledge of iodine nutrition in pregnant women was low, and as such initiatives to educate women of childbearing age on the importance of optimal iodine nutrition should be prioritized as a wider public health strategy to address global iodine deficiency [11–13]. Moreover, such findings could also support the food industry in ensuring adequate iodine in manufactured products to increase consumer consumption and thereby iodine intake, particularly given the changes in consumer trends e.g., increased rise of veganism and consumption of plant-based alternatives that are lower in iodine content [7]. Conversely, the use of iodised salt is a strategy that warrants further investigation as to whether this would be the best approach to adopt within the UK, given the success of public health anti-salt campaigns against Cardiovascular disease (CVD) and other co-morbidities [34]. Further, although the overall focus of this review is on pregnant women who are classified as at risk of iodine deficiency, consideration should also be given to sub-groups of the population, who may be at risk of excess iodine intake particularly if consuming rich dietary sources such as kelp [35]. Evidently, these findings reiterate that a broader strategy, understanding the changes in consumer and social trends, needs to be identified in helping to increase the iodine nutrition of both pregnant women, and women of childbearing age, especially given the impact on the cognitive outcomes of offspring.

As outlined in the exclusion criteria, systematic reviews and meta-analyses were excluded from this review, although the authors did hand-search relevant bibliographies. However, the authors feel it is important to highlight the recent findings of work published by both Dineva et al.*,* (2020) and Levie et al.*,* (2019;2020) [36–38]. Dineva et al.*,* (2020) published a systematic review and meta-analysis, focused on iodine supplementation, maternal/infant thyroid function, and inclusion of studies only within the mildly moderately iodine deficient range (50-149 μg/l) [36]. Dineva et al.*,* concluded that more focus on maternal intra-thyroidal stores should be considered with regards to iodine supplementation and advice to pregnant women [36]. Although our systematic review did not focus on iodine supplementation, the findings of Dineva’s work support the overall results of this review, whereby renewed focus on policy relating to iodine intake during pregnancy e.g., dietary choices and education, alongside focus on markers of iodine status such as maternal intra-thyroidal stores should be encouraged [21]. Moreover, the findings from Levie et al.*,* (2019;2020) who conducted meta-analyses, supports this focus on markers of iodine status, including free thyroxine (fT4), alongside the use of current urinary iodine assessments, particularly in relation to child IQ assessed [37–39].

The strength of this review highlights the importance of both urinary iodine assessment (UIC and/or ICr) alongside dietary intake data during pregnancy in relation to assessing relationships to cognitive outcomes in offspring, with the coherence between these two measurements within individual studies, supportive of future research. However, although the studies included in this systematic review yielded relevant results for the scope of this research, the type of studies included were of a prospective cohort/longitudinal design, coincidentally, following application of the inclusion/exclusion criteria aforementioned. Thus, other study types such as Randomized Control Trial’s (RCTs) are excluded from this review, which may provide a slightly biased/skewed approach to the overall findings, and thereby making it impossible to define cause/effect. As discussed in this review, there are limitations of both types of urinary iodine measurements (UIC and/or ICr) as a biomarker and in obtaining accurate dietary assessments which constrain the assessment of the mother’s iodine nutrition. Thus, future work should focus on assessing iodine intake and status through a combination of both dietary and biochemical means with the possibility to reflect the preconception period by using longer term markers of thyroid function such as thyroid stimulating hormone (TSH) or thyroxine (T4). In tandem with the recent work by Dineva et al.*,* (2020), concluding that there is insufficient good-quality evidence to support current recommendations for iodine supplementation during pregnancy, renewed focus should be on optimizing the dietary choices available e.g., milk/dairy produce alongside increased educational awareness which might prove more beneficial than supplementation independently [36]. Further, identification of the most robust cognitive tests to implement should also be reviewed, alongside the most suitable age to assess offspring and potential neurodevelopmental outcomes related to maternal iodine status and intake. The initial screening of abstracts was conducted by one reviewer (AM) and as such the authors acknowledge this as a potential limitation, however, the screening of full article texts and thereby the included studies within this review were independently screened by three of the authors as outlined (AM, MD & AY).

The studies included in this review support existing research into the crucial role iodine plays in foetal neurodevelopment, particularly deficiency leading to poorer cognitive outcomes. Future work not only needs to promote the importance of adequate iodine consumption in the preconception stages and throughout pregnancy but also needs to increase iodine awareness and knowledge as part of wider public health strategies to mitigate iodine deficiency.

## Conclusion

In conclusion, findings from this review indicate that the majority of studies classified pregnant women as mild-moderately iodine deficient based on urinary iodine assessment (UIC and/or ICr) or dietary intakes, with subsequent neurodevelopment implications identified in offspring. Although a considerable number of our studies did not report an association with neurodevelopmental outcomes, these findings are supportive of ensuring adequate dietary iodine intakes and urinary iodine monitoring throughout pregnancy. When viewed together, dietary intakes appear to indicate a more robust association with cognitive outcomes than urinary iodine measures independently. Therefore, future work is needed respecting the usefulness of urinary iodine assessment (UIC and/or ICr) as an indicator of deficiency whilst emphasizing dietary intakes owing to the identified link to longer-term consequences such as offspring neurodevelopmental outcomes of iodine nutrition.

## Supplementary Information

**Additional file 1.** Keywords/ search terms for dietary determinants of iodine nutrition during pregnancy on neurodevelopment.

## Data Availability

Data sharing is not applicable to this article as no datasets were generated or analysed during the current study.

## Abbreviations

ADHD: Attention Deficit Hyperactivity Disorder
ASD: Autism Spectrum Disorder
ASQ: Norwegian version of the Ages and Stages Questionnaire
BSID-III: Bayley’s Scale of Infant Development, third edition.
BRIEF-P: Behaviour Rating Inventory of Executive Function - Preschool Version
CBC: Child Behaviour Checklist
CCCS: The Children’s Communication Checklist-short
CI: Confidence Intervals
CVD: Cardiovascular Disease
EYFS: Early Years Foundation
FFQ: Food Frequency Questionnaire
HPLC: High Performance Liquid Chromatography
ICr: Iodine Creatine ratio
ICPMS: Inductively Coupled Plasma Mass Spectrometry
IQ: Intelligence Quotient
KS1: Key Stage One
MSCA: McCarthy Scales of Children’s Abilities
NS: Non-Significant
NAPLAN: National assessment program-Literacy and Numeracy
NOS: Newcastle Ottawa Scale
PRISMA: Preferred Reporting Items for Systematic Reviews and Meta-Analyses
RCT: Randomized Control Trial
SARIS: Student Assessment and Reporting Information System
T3: Triiodothyronine
T4: Thyroxine
TBG: Thyroxine Binding Globulin
TSH: Thyroid Stimulating Hormone
UNICEF: United Nations Children’s Fund
USI: Universal Salt Iodisation
WISC: Wechsler Intelligence Scale for Children
UIC: Urinary Iodine Concentrations
WHO: World Health Organization
