# Supplementary material for: Associations between maternal urinary iodine assessment, dietary iodine intakes and neurodevelopmental outcomes in the child: a systematic review
Source: Thyroid Res. 2021 Jun 7;14:14. doi: 10.1186/s13044-021-00105-1 (PMC8182912; doi:10.1186/s13044-021-00105-1)
Supplement: Supplementary file 1 — Additional file 1. Keywords/ search terms for dietary determinants of iodine nutrition during pregnancy on neurodevelopment. [file 13044_2021_105_MOESM1_ESM.docx]

**Additional File 1:**

***Keywords/ search terms for dietary determinants of iodine nutrition during pregnancy on neurodevelopment:***

EMBASE®

“diet” or “diet*.mp”

AND “iodine” or “iodine.mp”

AND “pregnancy” or “pregnant woman” or “maternal nutrition” or “mother” or “(pregna* or gestat* or impregna* or gravidness or intrauterine or childbearing or childbearing or maternal or mother*).mp

AND “nerve cell differentiation” or “cognition”

MedLine®

“diet” or “diet*”

AND “iodine” or “iodine.mp”

AND “pregnancy” or “pregnant women” or “(pregna* or gestat* or impregna* or gravidness or intrauterine or childbearing or childbearing or maternal or mother*)” or “Maternal Nutritional Physiological Phenomena” or “Mothers”

AND “brain” or “cognition” or “(brain or neurodevelop* or neurocognit* or cognit*).mp”

Web of Science®

(diet*)

AND (iodine)

AND ((pregna* or gestat* or gravidness or intrauterine or childbearing or “childbearing” or maternal or mother))

AND ((brain or neurodevelop* or neurocog
